# Bilateral optic neuropathy as first neurological sign of Central Nervous System (CNS) involvement in indolent chronic lymphocytic leukemia

**DOI:** 10.1007/s10072-025-08118-6

**Published:** 2025-03-25

**Authors:** Paola Ciasca, Sebastiano Crisafulli, Ludovica Gargiulo, Stefania Bianchi Marzoli, Massimo Magagnoli, Marco Moscatelli, Elena Anghileri

**Affiliations:** 1 Neuro-Ophthalmology Center and Ocular Electrophysiology Laboratory, Scientific Institute Capitanio Hospital, IRCCS Istituto Auxologico Italiano, Milan, Italy; 2 Neuroimmunology and Neuromuscular Diseases Unit, Fondazione IRCCS Istituto Neurologico Carlo Besta, Via Celoria, 11, 20133 Milan, Italy; 3 Department of Hematology and Clinical Oncology, Humanitas Research Hospital Rozzano, 20089 Milan, Italy; 4 Neuroradiology Unit, Fondazione IRCCS Istituto Neurologico Carlo Besta, Via Celoria, 11, 20133 Milan, Italy; 5 Neuro-Oncology, Fondazione IRCCS Istituto Neurologico Carlo Besta, Via Celoria, 11, 20133 Milan, Italy

Dear editor in Chief

Chronic lymphocytic leukemia (CLL) is the most common leukemia in adulthood, it affects more than 200 000 people and it is associated with approximately 4410 deaths in the US annually [1].

Possible leukemic infiltration of the skin, lung, pleura, kidney and gastro-intestinal tract by CLL cells is already known. Central nervous system (CNS) involvement in patients with CLL have an incidence of 0.8–2% [2] and includes manifestations such as ataxia, cranial nerve palsies, dementia, diplopia and confusion.

As recently reported by Liu et al. [3], only 16 cases of leukemic infiltration of the optic nerve as initial manifestation of CNS involvement are described so far, and for some of them brain Magnetic Resonance (MR) was described as negative.

We reported a new case of infiltrative optic neuritis (ON) in a CLL.

## Case report

A 53-year-old woman developed progressive bilateral blurry vision and bilateral eye pain. She had no other signs or symptoms except for a few cervical enlarged lymph nodes, present from two years, and stable. The patient had a diagnosis of CLL stage A (Binet staging) two years earlier based on peripheral blood lymphocyte typing, she was not treated with any specific therapy and underwent regular follow-ups.

One month after symptoms onset, the neuro-ophthalmological examination showed diminished Visual Acuity (0.9 in right eye and 0.8 in left eye), the anterior segment was normal as well as ocular pressure. Fundoscopic examination showed bilateral optic disc edema (Frisen Scale 2) confirmed by Optical coherence tomography that displayed elevated nerve fiber layer. Eye ultrasound confirmed the presence of bilateral papilledema with increased thickness of the optic nerve.

**Fig. 1:**
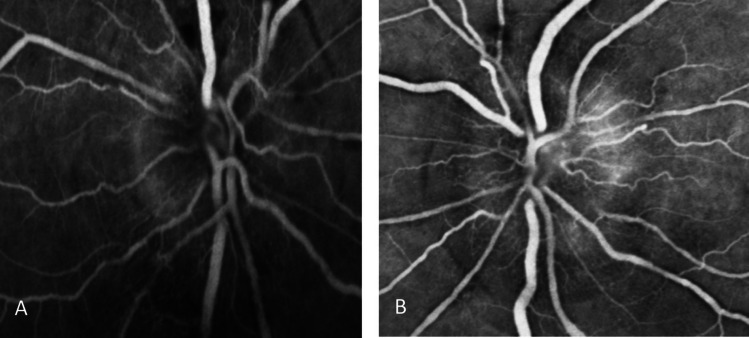
Ocular angiography shows late bilateral papillary leakage (**A**-**B**) and in right eye hypofluorescence from upper peripapillary preretinal hemorrhages (**A**)

**Fig. 2:**
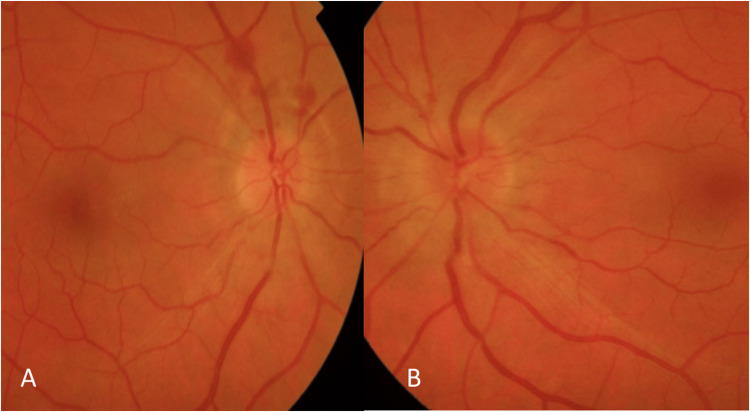
Fundus photography shows bilateral optic disc edema (Frisen Scale 3) **A**—**B** and upper peripapillary preretinal hemorrhages in right eye (**A**)

The ocular angiography showed late bilateral papillary leakage in right eye hypofluorescence from upper peripapillary preretinal hemorrhages (Fig. 1 – 2).

**Fig. 3:**
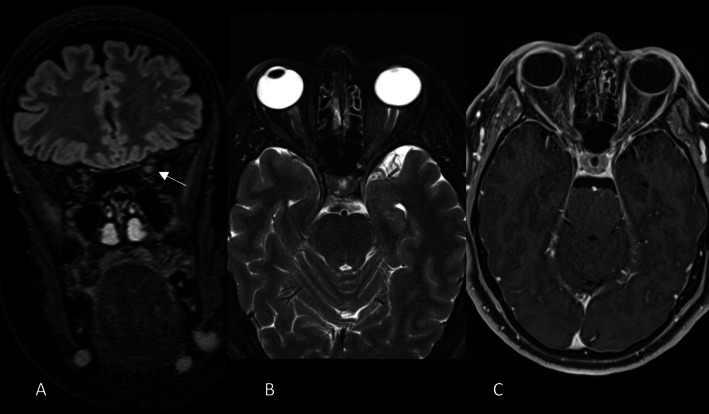
MR shows subtle hyperintensity along the optic nerve in FLAIR coronal images (arrow in **A**). No significant swelling of the nerves in T2 axial fat sat images (**B**) nor clear enhancement in T1 after gadolinium administration (**C**)

Then brain MR showed a subtle Fluid-attenuated *inversion* recovery (FLAIR) hyperintensity in the pre-chiasmatic tract of the left optic nerve. No significant swelling or enhancement of the optic nerve were seen (Fig. 3).

Only 4 months after the onset, she was referred for neurological evaluation (that documented no additional signs except to optic disc edema) and then she was hospitalized for complete diagnostic work-up.

Cerebral spinal fluid (CSF) analysis showed normal glucose, normal protein and the presence of 59 cells/mm^3^ White Blood Cells (WBC), with 97% of lymphocytes including 79.5% of abnormal CD5 + B*-*cell population at typing analysis, suggesting the cell B CD5 + lymphoproliferative disease. CSF-Anti EBV antibodies were negative. Complete blood count unremarkable except for elevated revealed WBC count (20,950 cells/mL) (normal: 4,500 − 10,000 WBC/mL) with 58.6% lymphocytes.

Cytometric analysis of peripheral blood showed 81.1% of CD19 + B Lymphocytes with regular shape and the following atypical immunophenotype: CD19 + , heterogenous CD20 + , CD5 + , CD23-/ + (30%, weak), CD79b + (intermediate intensity), CD81 + (weak), CD10-, CD38-/ + , FMC7-, kappa + (weak). Such data are compatible with CD5 + B cells lymphoproliferative disease.

Additional analysis as serological autoimmune profile and Anti-Aquaporin 4 (AQP4) antibodies resulted negative.

Optic nerve biopsy was not performed due to the positivity of CSF markers and the high probability of secondary visual impairment.

The patient started 1 g/day of intravenous methylprednisolone for 5 days, with immediate slight improvement of optic disc edema. No further tapering of steroid was performed.

She then underwent to systemic work-up, including total body Positron Emission *Tomography* (*PET*) *imaging* with 2-[(18)F]fluoro-2-deoxy-D-glucose (*FDG)* that showed lateral-cervical lymphnodal hypercaptation. A lymph node biopsy was then performed, and showed predominantly B lymphoid proliferation with Ki 67 value of 30%, consistent with CLL. The white cell count was 7200/uL and 1200/L for total blood cells and total lymphocytes respectively.

Based on the absence of histologic transformation in the bone marrow and in lymph nodes and the absence of biological and clinical negative prognostic factors systemic therapy was not started, and the patient continued with periodical stringent follow-up.

She did not complain any blurry vision and pain by the 3-month and 6-month follow up.

The following neuro-ophthalmological evaluation after 3 months of steroid therapy showed a clear improvement: in particular, optical disc edema resolved, associated with a complete disappearance of the preretinal hemorrhages; visual acuity remained stable. A further examination 6 months later confirmed the well-control of neurological alterations, showing visual acuity stability andster mild change, based on reduction of retinal nerve fiber layer and ganglionar cells at OCT analysis.

Further brain MR was stable compared to the exam five months earlier.

The last haematological evaluation did not show systemic disease progression.

## Conclusion

Differential diagnosis of bilateral optic neuropathy with optic disc edema can be challenging as it includes (in order of frequency) venous sinus thrombosis, idiopathic intracranial hypertension and tumors, infectious meningitis, leptomeningeal metastasis, arterial hypertension and meningitis [4]. Once the most frequent causes are excluded, it is mandatory to investigate alternative and rarest ones. Optic nerve involvement in lymphoproliferative diseases has been described both as a rare localization of disease recurrence and as the first manifestation of disease. Ophthalmic involvement was more frequently bilateral in CLL than in lymphoma or infections. Among the 16 cases described by Liu and colleagues [3], five were Rai Stage 0 and one was Rai stage 1 (equivalent to stage A by Binet staging system). The present case got a diagnosis of CLL stage A two years before the CNS involvement and was treatment-naive. We decided not to treat the patient immediately with chemo-immunotherapeutic treatment becouse the drugs commonly used in CLL do not cross the blood–brain barrier, and due to the rapid clinical response to steroid treatment, we prefer to wait before using BTK inhibitors.

In patients with known lymphoproliferative diseases presenting with visual symptoms, it is mandatory to search for optic nerves and CNS involvement as early diagnosis is crucial to preserve the visual function and to optimize disease staging and treatment.

No systematic reviews currently exist for risk factors of CNS involvement in patients with CLL. However, CNS involvement has not been correlated with the CLL disease stage, course, sex, age, immunophenotype, and high-risk chromosomal and molecular abnormalities [5].

Our case also confirms that CSF immunophenotyping could be a feasible alternative to nerve biopsy. Moreover, the steroid treatment was successful and the patient got functional ophthalmic remission with no other signs of CLL progression. Others besides steroids reported also optic nerve radiation therapy and intrathecal and/or systemic chemotherapy [3]. As largely showed in other context of relapsed/refractory CLL high-dose corticosteroids play a significant role in disease control; and canimprove blood flow on optic nerve head in patients with acute ON [6].

Given the rarity of the disease, and the absence of a shared specific treatment protocol, this report can help to define the diagnostic and therapeutic path of such clinical picture.

## Data Availability

For further inquiries can be directed to the first author or corresponding author.
